# Transgastric biopsy of a submucosal gastric tumour

**DOI:** 10.1259/bjrcr.20160009

**Published:** 2016-11-02

**Authors:** Joachim Kettenbach, Martin Mittendorfer, Irina Wimmer, Marcus Mader, Eva Taubenschuss, Eva Unfried, Gertrud Heinz

**Affiliations:** ^1^Institute of Medical Radiology, Diagnostic, Intervention, Karl Landsteiner University of Health Science, University Hospital St. Pölten-Lilienfeld, St.Pölten, Austria; ^2^Institute of Nuclear Medicine, Karl Landsteiner University of Health Science, University Hospital St. Pölten-Lilienfeld, St. Pölten, Austria; ^3^Internal Medicine 2, Karl Landsteiner University of Health Science, University Hospital St. Pölten-Lilienfeld, St. Pölten, Austria; ^4^Institute of Pathology, Karl Landsteiner University of Health Science, University Hospital St. Pölten-Lilienfeld, St. Pölten, Austria

## Abstract

Endoscopic biopsy is thought to be the best method to obtain biopsy samples of the gastrointestinal tract. In our case, however, an endoscopic forceps biopsy failed to confirm malignancy of an intramural gastric tumour. Since the tumour, about 4 cm in diameter, was well delineated on the CT scan, the patient was referred for a percutaneous CT-guided needle biopsy, which confirmed a gastrointestinal stromal tumour.

## Summary

**Figure 1.**
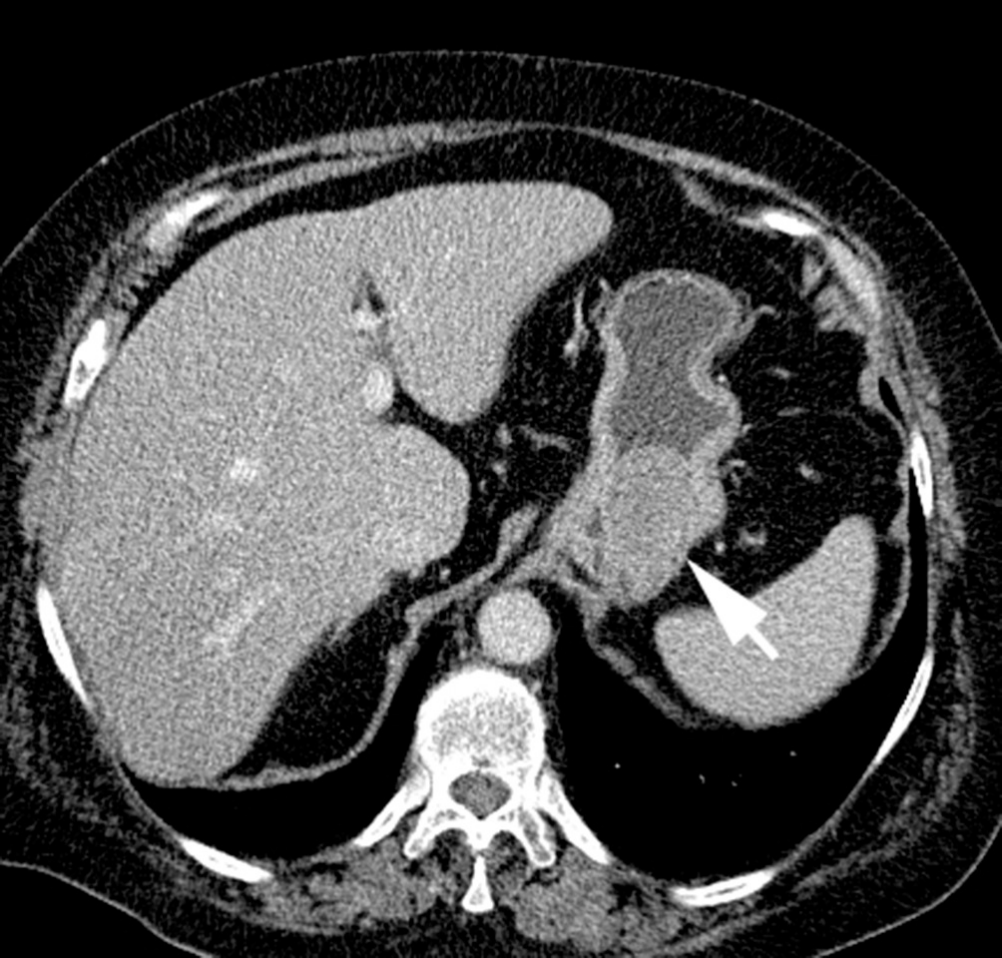
A nodular, contrast-enhancing gastric tumour, about 4 cm in diameter, is seen on the CT scan (arrow).

A 64-year-old female was referred to our hospital because of an endometrial cystic lesion showing up on MRI of the pelvis (not shown). Besides abdominal discomfort and arterial hypertension, there were no further relevant signs or symptoms, and her family history was unremarkable. As part of the evaluation, a contrast-enhanced CT scan of the abdomen was performed (slice thickness 4 mm, 90 ml Accupaque 350 mg J ml^−1^, scan delay 70 s post injection), which showed a calcified fibroid of about 5 cm diameter, few slightly enlarged pelvic lymph nodes and a nodular contrast-enhancing tumour within the gastric wall, about 4 cm at its largest diameter (Figure 1). The patient underwent hysteroscopy and curettage of the benign endometrial polyp.

**Figure 2.**
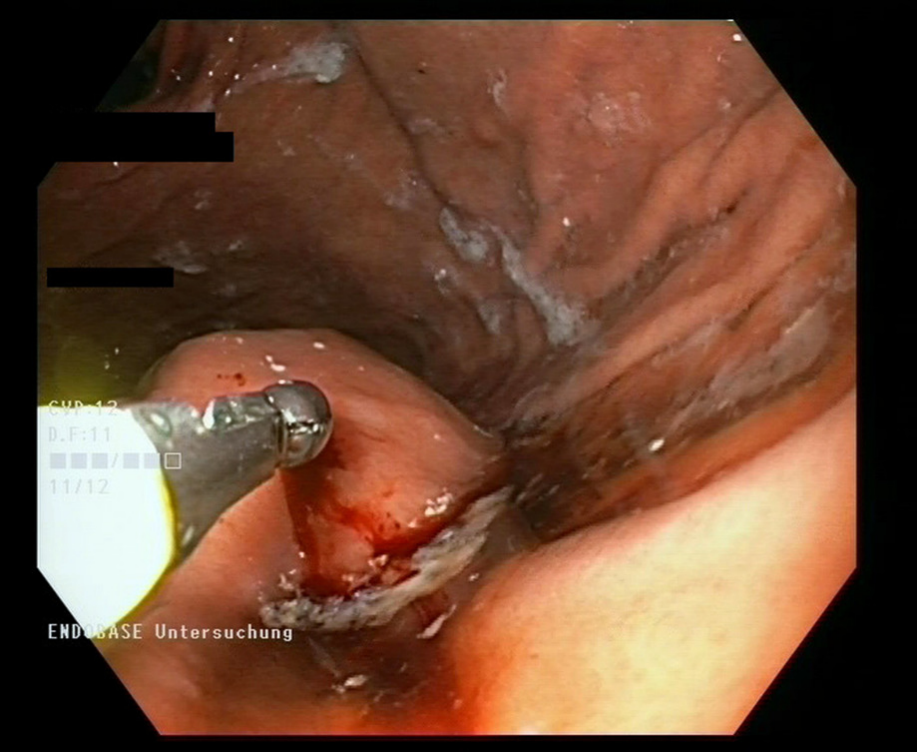
Endoscopic view of the submucosal gastric tumour during forceps biopsy.

Subsequently, gastroscopy demonstrated a bulging submucosal tumour in the fundal area (Figure 2). Forceps biopsy obtained from the bulging lesion demonstrated signs of mild chronic gastritis and foveolar hyperplasia, but no structure was found that would explain the bulging lesion, nor were malignant cells obtained. 2 weeks later, an ^18^F-fludeoxyglucose positron emission tomography (325 MBq) was performed, but no hypermetabolic structures were found in either the stomach or the pelvic area. However, an ^18^F-fludeoxyglucose-negative signet-ring cell carcinoma or a gastrointestinal stromal tumour (GIST) was still considered as a differential diagnosis. The origin of the tumour was still unclear, but as the tumour was well delineated on the contrast-enhanced CT scan, a percutaneous CT-guided needle biopsy was considered to obtain valid histological information. We were encouraged by the successful results published by others,^1–4^ and a biopsy was scheduled 4 weeks after the initial diagnosis.

**Figure 3.**
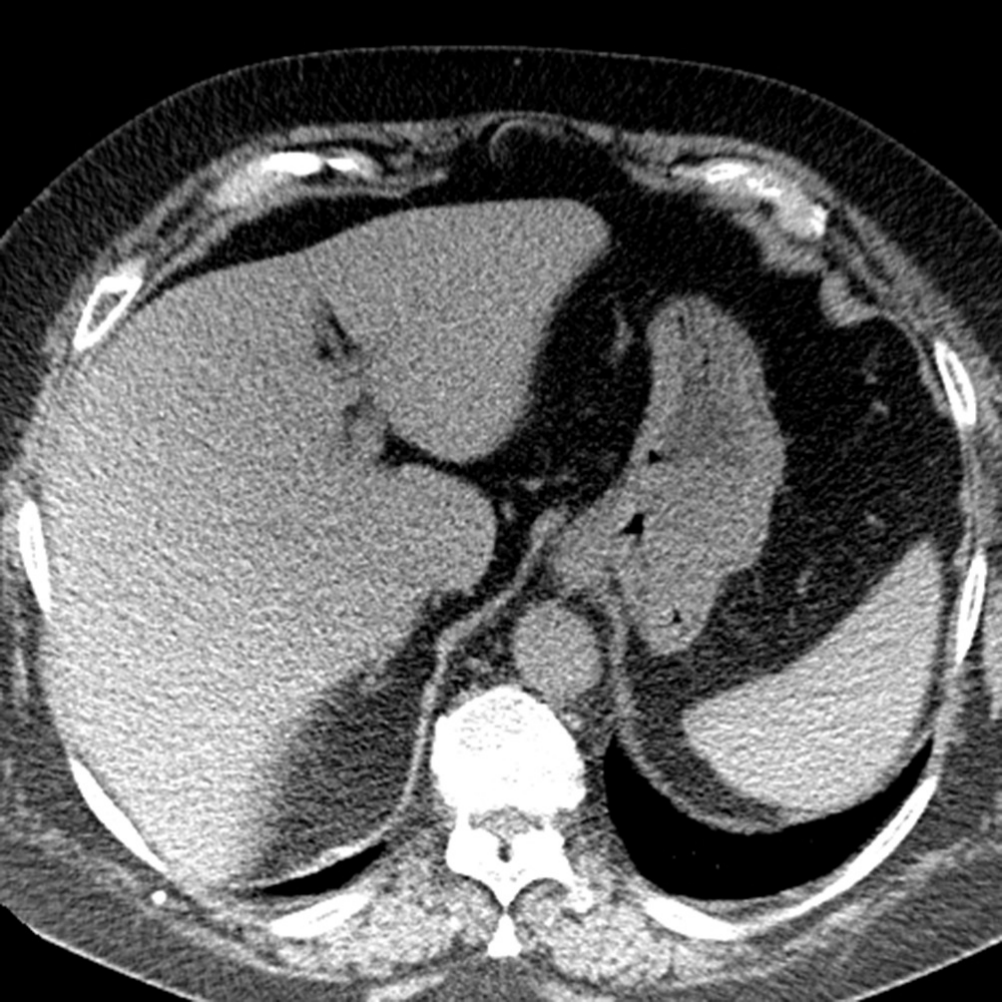
On unenhanced CT scan, the gastric tumour is barely delineated from the surrounding structures and seems to be inaccessible for biopsy through the anterior abdominal wall.

**Figure 4.**
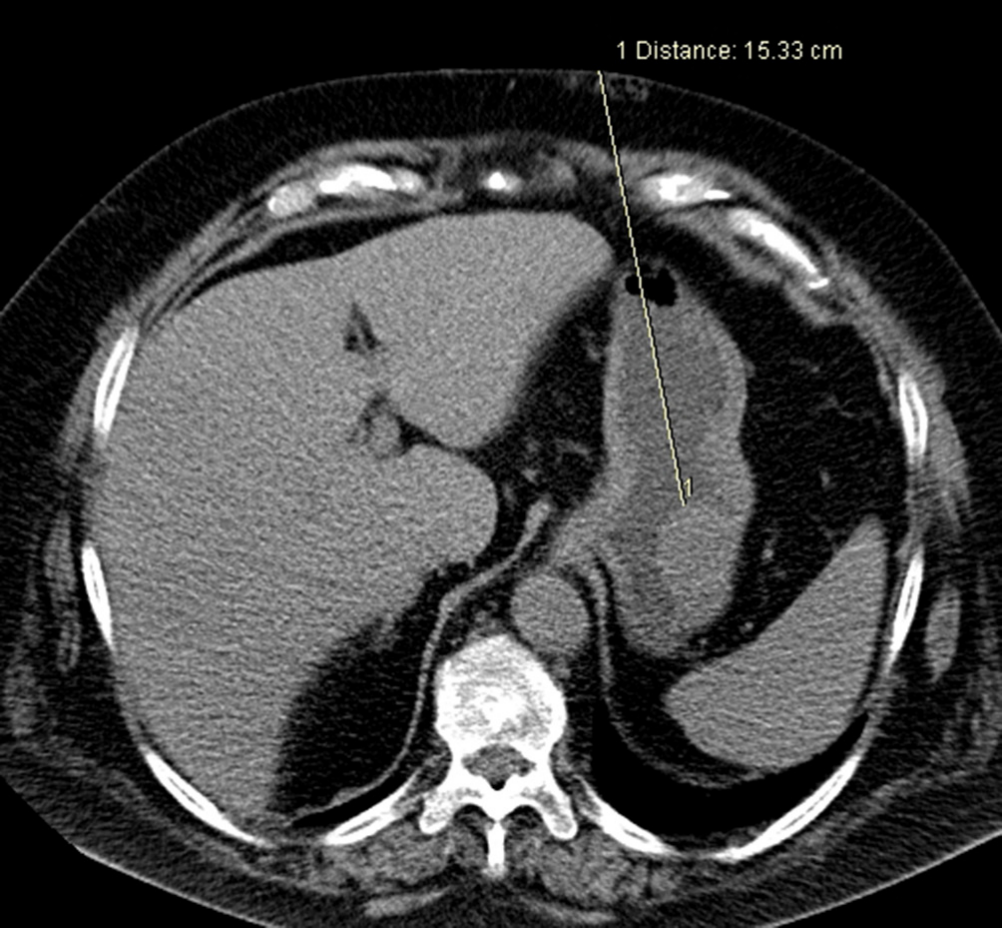
Expansion of the gastric lumen by having the patient swallow effervescent granules with small amounts of water improved delineation of the gastric tumour.

The patient gave oral and written informed consent before the biopsy. All oral feeds and fluids were stopped overnight. The patient was placed in the supine position, with both arms elevated above the head. First, an unenhanced CT scan (slice thickness 3 mm) of the upper abdomen was obtained during shallow breathing to localize the gastric tumour and plan the best entry site. However, the gastric lumen was partially collapsed and the tumour was barely distinguishable from secretions inside the gastric lumen (Figure 3). In order to improve the delineation of the tumour and expand the gastric lumen, we asked the patient to swallow a spoonful of effervescent granules (European Pharmacy, Vienna, Austria) with small amounts of water to wash the granules down into the stomach. Following this, another unenhanced CT scan (Figure 4) was obtained that showed the gastric lumen was now slightly expanded and the tumour was accessible through a transgastric route from the epigastrium. The distance from the skin to the surface of the tumour was 15.3 cm.

**Figure 5.**
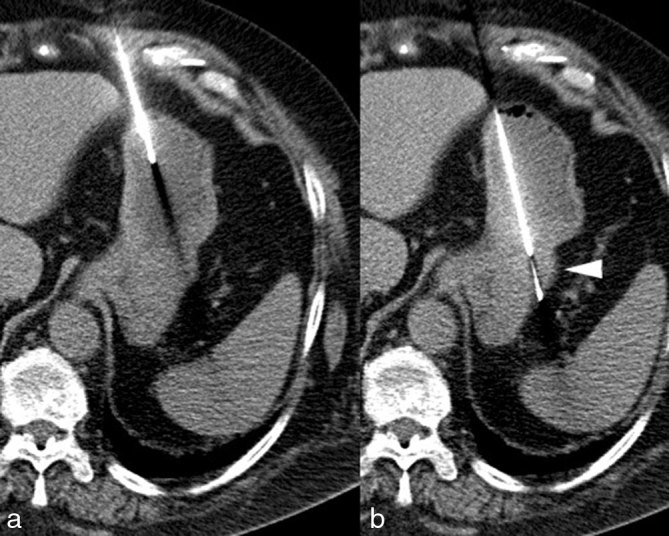
First, the tip of a 17 G puncture needle (length 17 cm) was pushed through the anterior gastric wall, heading to the tumour (a). Next, the 18 G biopsy needle was inserted and the biopsy chamber was exposed within the tumour (right, white arrow). Subsequently, a spring-loaded mechanism released the outer hull of the biopsy needle to cut off a specimen from the tumour and capture the biopsy sample within the needle sheath (b).

The epigastrium was then prepared in a sterile manner and local anaesthesia (1% lidocaine) was applied down to the peritoneum and throughout the anterior gastric wall. The white line shows the distance between the skin entry point and the surface of the gastric tumour along the proposed biopsy path. To enable further needle insertion, we then used a CT scan protocol dedicated for biopsy, in which each scan acquired in between the biopsy steps consisted of a series of three slices (thickness 6 mm). The triplet of scan slices was repeated before and after each interventional step while we moved in and out of the scanner room. After a small skin incision was made, a 17 G puncture needle of length 17 cm (TruGuide, Bard, Tempe, AZ) was gradually inserted during breath-hold through the anterior gastric wall until the tip of the puncture needle was just at the tumour surface. The inner stylet of the puncture needle was then removed and a semiautomatic 18 G cutting biopsy needle (20 cm long, Somatex, Teltow, Germany) was inserted. The position of the exposed 20 mm biopsy chamber of the sharp metal stylet within the tumour was confirmed (Figure 5) just before the core biopsy sample was obtained. After biopsy, the puncture needle was removed and the tissue sample obtained was put into 4.5% formaldehyde solution for histological examination.

## Outcome and follow-up

CT scans performed immediately after the biopsy excluded any bleeding and pneumoperitoneum. The patient was moved back to the ward with bed rest and no oral feeding or liquids for another 3 h. Vital signs were closely monitored for signs of peritonitis, which might indicate leakage of gastric contents. The patient did well and was discharged the following day. Histological examination revealed a C-kit-positive (DOG1, CD34) GIST with a low Ki67 proliferation rate (< 10%); however, the small sample size showed < 1 mitotic figure per 10 high power field (HPF) and thus did not allow accurate grading of the tumour, which requires evaluation of 50 HPF. Based on a consensus decision of the internal tumour board, the patient underwent a successful laparoscopic resection of the gastric tumour 7 weeks after the initial biopsy and is doing well since then. Post-surgical histopathological examination confirmed the primary histological diagnosis and demonstrated a low-risk intramural GIST (< 5 mitosis per 50 HPF). Based on guidelines, no further therapy was required.^5^

## Discussion

Endoscopy is considered the gold standard for evaluating tumorous lesions of the gastrointestinal (GI) tract. In some cases, however, forceps biopsy of an endophytic tumour can be challenging and may fail to provide reliable histology.^2^ An endoscopic ultrasound-guided fine needle aspiration may offer a diagnostic accuracy of 60–90% depending upon the site, but is associated with limited diagnostic accuracy for GISTs.^4^ In these cases, larger calibre cutting needles for endoscopic biopsy are recommended^6^ but these were not available in our case. We considered percutaneous CT-guided needle biopsy to be suitable, which is a well-recognized method for biopsy of many solid organs, but is less often used for the biopsy of GI lesions. To our knowledge only the following authors described the role and safety of percutaneous gastric biopsy small bowel or colon,^1–4^ although a transgastric access is well established for percutaneous gastrostomy.^7^ Before biopsy, non-invasive assessment of the gastric wall is mandatory. While some authors have recommended up to 1500 ml of water or flavoured methylcellulose as an oral contrast and 20 mg of intravenous scopolamine to expand the gastric wall,^8^ we used effervescent granules administered orally.

In order to minimize the risk of haemorrhage, the inferior epigastric artery, which is well seen even on an unenhanced CT scan, should be avoided, as it courses through the junction of the medial two-thirds and lateral one-third of the rectus muscle. To avoid the risk of displacing the gastric wall when pushing the puncture needle into the stomach, a gastropexy device [a 17 G needle preloaded with a Cope suture anchor (Cook Inc., Bloomington, IN)] may be used.^7^ In any case, the puncture should be made with a brief, deliberate thrust so as not to push the anterior gastric wall away from the anterior abdominal wall. While Perez-Johnston et al^4^ recommended obtaining a biopsy sample from the thickest area of a GI lesion, thus avoiding traversing the wall into the lumen, a gastric lesion located in the fundus or the lesser curvature may require a transgastric approach, as in our case.

In general, percutaneous needle biopsy has a reported overall complication rate of approximately 8%, although 2% require further intervention,^4^ whereas others^9^ reported a bleeding rate of 3%, and a risk of pneumocolon and peritonitis of 0.3%, following transgression of the bowel with an 18 or 19 G needle. However, bowel transgression can lead to possible abscess formation and should be avoided in any case.

Interestingly, a significantly higher bacterial load in the stomach was observed in patients using a proton pump inhibitor; this emphasizes the need to cease the use of proton pump inhibitors prior to transgastric interventions to avoid infection.^10^ Similar to percutaneousgastrostomy, limited percutaneous access to the stomach (*e.g.* massive hepatosplenomegaly or interposed colon) and uncorrectable coagulopathy are considered contraindications for percutaneous transgastric biopsy.

## Conclusions

Endoscopic biopsy is thought to be the best method for obtaining valid histological information from the GI tract. However, in specific cases where traditional endoscopic biopsies are not feasible or are non-diagnostic, an image-guided percutaneous biopsy of an intramural gastric tumour may be considered an effective and safe technique with which to obtain a valid histological diagnosis; the morbidity and cost of a surgical biopsy under anaesthesia can thus be avoided.^2^

## Learning points

1. Percutaneous biopsy of a gastric tumour is feasible, safe and effective.
2. Percutaneous gastric biopsy should be offered if endoscopic biopsy fails.
3. Expanding the gastric lumen by having the patient swallow effervescent granules improves the delineation of a bulging gastric tumour.

## Consent

Written informed consent for this case to be published (including images, case history and data) was obtained from the patient.
